# A Multi-Resolution Mode CMOS Image Sensor with a Novel Two-Step Single-Slope ADC for Intelligent Surveillance Systems

**DOI:** 10.3390/s17071497

**Published:** 2017-06-25

**Authors:** Daehyeok Kim, Minkyu Song, Byeongseong Choe, Soo Youn Kim

**Affiliations:** 1 Department of Semiconductor Science, Dongguk University-Seoul, Seoul 04620, Korea; dh7423@dongguk.edu (D.K.); mksong@dongguk.edu (M.S.); 2 Department of Information and Telecommunication Engineering, Dongguk University-Seoul, Seoul 04620, Korea; bchoe@dongguk.edu

**Keywords:** CMOS image sensor, fixed pattern noise, intelligent surveillance system (ISS), low power consumption, multi-mode pixel resolution, two-step single-slope ADC

## Abstract

In this paper, we present a multi-resolution mode CMOS image sensor (CIS) for intelligent surveillance system (ISS) applications. A low column fixed-pattern noise (CFPN) comparator is proposed in 8-bit two-step single-slope analog-to-digital converter (TSSS ADC) for the CIS that supports normal, 1/2, 1/4, 1/8, 1/16, 1/32, and 1/64 mode of pixel resolution. We show that the scaled-resolution images enable CIS to reduce total power consumption while images hold steady without events. A prototype sensor of 176 × 144 pixels has been fabricated with a 0.18 μm 1-poly 4-metal CMOS process. The area of 4-shared 4T-active pixel sensor (APS) is 4.4 μm × 4.4 μm and the total chip size is 2.35 mm × 2.35 mm. The maximum power consumption is 10 mW (with full resolution) with supply voltages of 3.3 V (analog) and 1.8 V (digital) and 14 frame/s of frame rates.

## 1. Introduction

Due to their low cost, low power consumption, and easy integration of analog/digital processing functions on a chip, CMOS image sensors (CIS) have driven the market over charge coupled device (CCD). The achievement of such inherent characteristics of CIS is highly desirable for intelligent surveillance systems (ISS) [1,2]. The CIS in ISS observes an environment and then transmits data to monitors equipped with a video recorder for security purposes. Recently, for home and public surveillance, ultralow-power CIS [3,4,5] that are battery-operated have widely been used owing to their accessibility and easy installation. In addition, since the CIS in ISS is required to always be turned on, even when there are no security-related events (called peace mode), lowering image resolution during peace mode can further reduce power consumption. Therefore, in order to make CIS in ISS more power-efficient, configurable resolution depending on whether security-related events occur or not is necessary to select low-resolution mode and high-resolution mode. 

In this paper, in order to make images configurable, the proposed CIS supports more sub-sampling ratios from 1, 1/2, 1/4, 1/8, 1/16, 1/32, and 1/64 resolution mode. Smaller resolution modes lead to lower power consumption by disabling some parts of peripheral circuits (such as row control block and column parallel ADC [6,7,8]). In addition, for the high quality mode when security-related events happen, we improve the performance of a two-step single-slope analog-to-digital converter (TSSS ADC). The nonlinearity and column fixed-pattern noise (CFPN) of TSSS ADCs mainly come from parasitic capacitances on the input node of comparators in correlated double sampling (CDS) located in each column. Since the parasitic capacitances in comparators of conventional TSSS ADC affect the slope of ramp signals during coarse ADC and fine ADC operation, we separate the input nodes of comparators for ramp signals for coarse ADC and fine ADC, respectively by using 4-input comparators, leading to the reduction of parasitic capacitances in our proposed comparators. The contents of the paper are as follows: in Section 2, an intelligent surveillance system and a pixel sub-sampling technique are introduced. The circuit design and implementation are discussed in Section 3. Measurement results and conclusions are summarized in Section 4 and Section 5, respectively.

## 2. Intelligent Surveillance Systems

**Figure 1:**
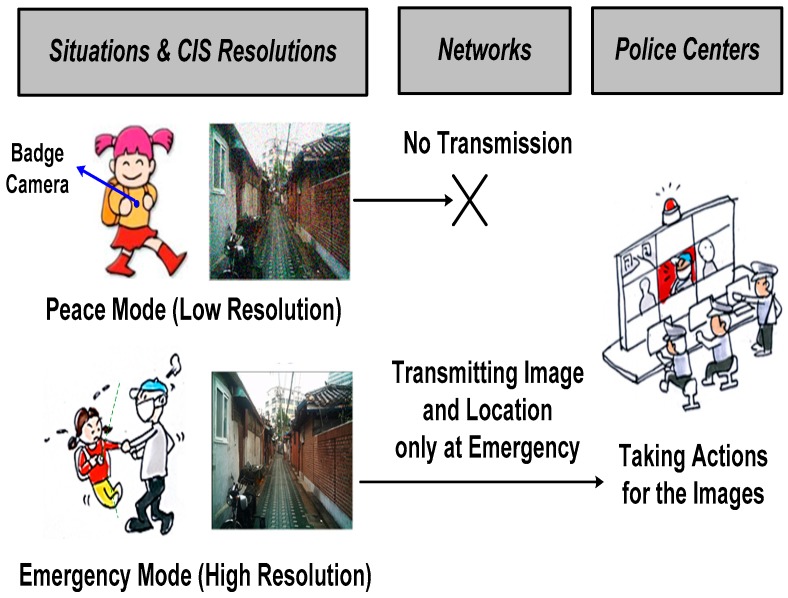
A brief explanation of an intelligent surveillance system (ISS).

Figure 1 provides a brief illustration of a battery-powered intelligent surveillance system (ISS). The CIS in the ISS operates with two different modes: low-resolution mode and high-resolution mode. In the low-resolution mode case, there are no security-related events, and it is called peace mode. In this mode, CIS in the surveillance system is not required to capture high-resolution images. However, when an emergency situation happens, the images should be captured with high-resolution mode (called emergency mode) to transmit the detailed information about the event, such as captured images and locations to the nearest police center. Since the battery-powered CIS should always be turned on to monitor whether security-related events occur or not, the low-power performance is necessary. Therefore, in order to reduce the total power consumption of CIS in ISS, we suggest sub-sampling method during reading out the image data in low-resolution mode.

**Figure 2:**
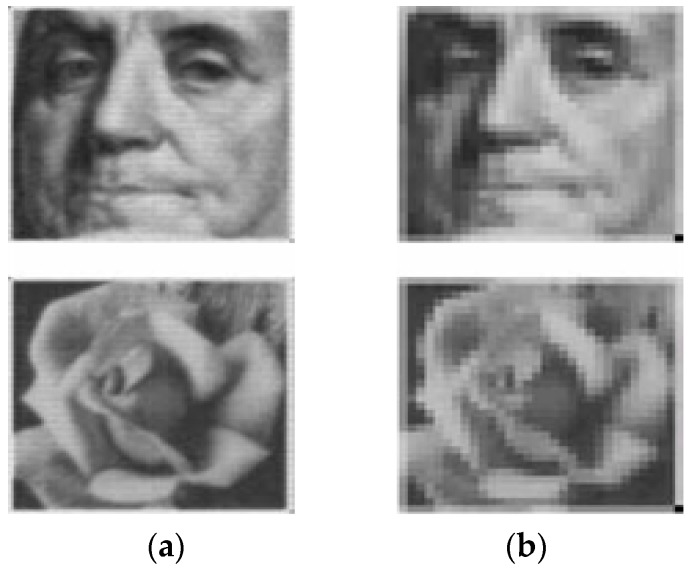
Pixel sub-sampling technique (**a**) high-resolution mode; (**b**) low-resolution mode.

There are two ways to make the image resolution configurable to achieve low-power CIS during low-resolution mode: (1) sub-sampling and (2) binning [9,10]. Sub-sampling methods skip multiple pixels and ADCs during reading out the image data, leading to power-savings proportional to the sub-sampling ratio used. On the other hand, binning methods average or adds multiple pixels with additional circuits for the functions in the pixel array or ADCs, leading to extra power consumption. Even though the binning method results in a wide dynamic range while the captured images with sub-sampling have low resolution, as shown in Figure 2, we choose the sub-sampling method to maximally reduce power consumption during the peace mode of ISS. It should be noted that we discuss about the sub-sampling of a monochrome sensor in this paper.

## 3. Circuit Description

### 3.1. Structure of the CMOS Image Sensor (CIS)

**Figure 3:**
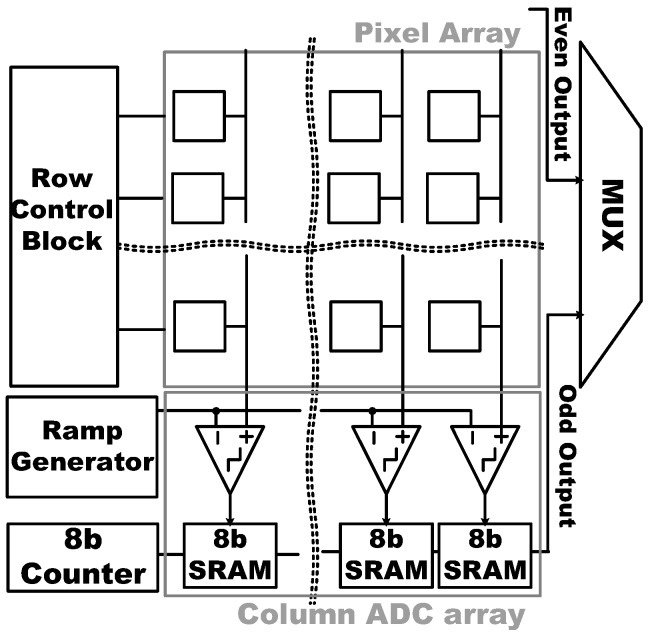
A block diagram of the proposed CIS.

Figure 3 shows the block diagram of the proposed CIS in this paper. The CIS structure consists of a pixel array (176 × 144), 8-bit column-parallel analog to digital converter (ADC) including comparators in the correlated double sampling (CDS), 8-bit static random access memories (SRAMs), 8-bit counter, digital timing control blocks (=row control block), and multiplexer (MUX) for data read-out. The pixel converts the amount of light into the corresponding voltage, which is the input for the ADC where the pixel output voltage is transformed into a digital code. The row control block controls the timings for pixel operation, ADC operation, and data read-out, respectively. In addition, pixel sub-sampling method makes image resolution configurable to achieve low-resolution with low-power mode and the sub-sampling ratio can be chosen from 1/2 to 1/64. The detailed operation principle of the proposed ADC will be discussed in the following sections.

### 3.2. Multi-Mode Pixel Resolution

By adopting configurable resolution features, CIS can activate different resolution modes such as either in high-resolution or low-resolution modes. Sub-sampling method is used for variable-resolution operation (such as 1, 1/2, 1/4, 1/8, 1/16, 1/32, and 1/64), which only processes the number of pixels required for the given resolution. In low-resolution mode, the number of pixels to be processed is smaller, resulting in reduced average power consumption for a given time span. For the configurable resolution feature, CIS consists of a single unit of 8 × 8 pixels consisting of 4-shared 4T-active pixel sensors (APS), and 4-shared column ADC. 

**Figure 4:**
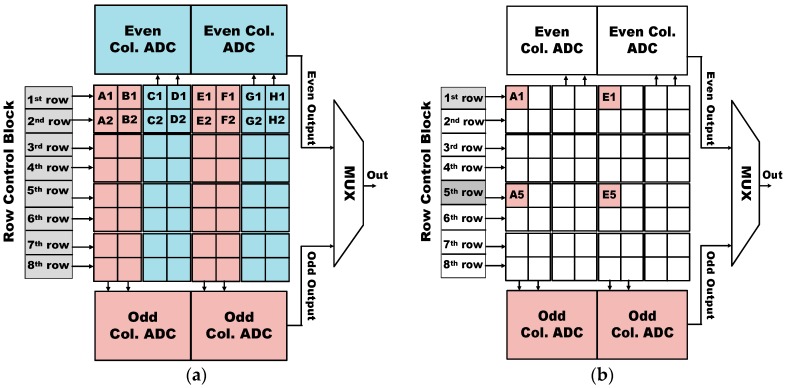
Simplified principle operation of multi-mode pixel resolution with 16 × 16 pixel array and ADC array for (**a**) high resolution mode and (**b**) 1/16 resolution mode.

Figure 4a shows the operation of high-resolution mode, where odd/even ADC column arrays and row control arrays are all operated. It should be noted that a column ADC in this work requires the layout pitch as same as the width of four pixels as shown in Figure 4a. Therefore, the outputs of A1 and B1 pixels are connected to odd column ADC, while those of C1 and D1 pixels are connected to even column ADC. After reading out the first row’s pixel outputs, the second row’s pixel outputs are read out in consecutive order with row control block. On the other hand, for the operation of 1/16 mode, one pixel of every four rows and columns is only turned on. As shown in Figure 4b, since both A1 and E1 pixels are connected to odd column ADC, even column ADC can be turned off, leading to the reduction of the power consumption of ADC by half. Further, the row control block for the first and fifth row only is need to be operated, while those for other rows can be turned off to save power consumption. Finally, for the case of 1/64 mode, since only A1 pixel is turned in 16 × 16 pixel array, half of odd column array and seven row control blocks can be turned off, leading to the maximum reduction of power consumption during the low-power mode of ISS.

### 3.3. Proposed Two-Step Single-Slope ADC (TS SS-ADC)

**Figure 5:**
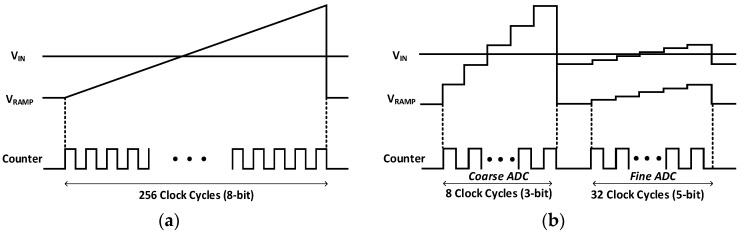
Operation principle of (**a**) SS ADC and (**b**) TSSS ADC.

Among the column-parallel ADC architectures, single-slope (SS) ADC is widely used for commercial high-resolution CMOS image sensor applications. However, SS ADC is not power efficient, compared to other ADC schemes, because ADC operation period is exponentially increased with ADC resolution. As for an example of an 8-bit SS ADC, at least 2^8^ cycles (=256 cycles) is required to finish ADC operation. Figure 5a show the operation of 8-bit SS ADC. Each comparator in a column compares V_RAMP_ (from ramp generator as shown in Figure 3) and V_IN_ (from APS). When V_RAMP_ starts, 8-bit counter is simultaneously enabled to count output code. Once V_RAMP_ exceeds the V_IN_, the output of comparator is flipped and the counter value at that moment is read out.

In order to reduce the time for ADC operation, two-step single-slope (TSSS) ADC has been recently reported in [11]. TSSS ADC requires only 2^M^ + 2^N^ cycle to finish ADC operation with M + N-Bit of coarse and fine ADCs. As shown in Figure 5b, for 8-bit TSSS ADC, coarse ADC converts 3-bit of the most significant bits (MSB) and fine ADC converts 5-bit of the least significant bits (LSB). During the coarse ADC operation, all comparators use V_RAMP_ that covers full scale of V_IN_ to find upper 3 MSB of the overall ADC. After then, each column selects one of the multiple ramp generators depending on the coarse ADC outputs for fine ADC operation that leads to the output of the fine ADC corresponding to the lower 5 bits of the overall ADC. Finally, TSSS is required to 2^3^ + 2^5^ cycles (=40 cycles) for ADC operation that is 216 cycles less than that of SS ADC. 

**Figure 6:**
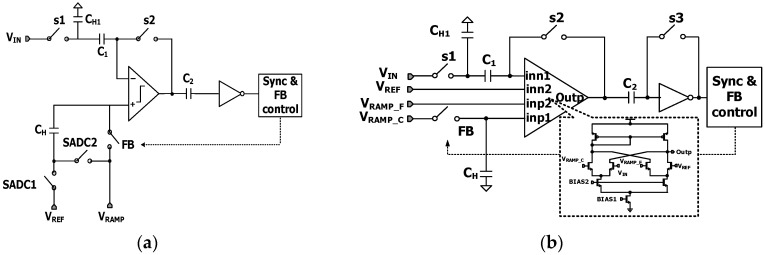
Circuit diagram of (**a**) conventional TSSS ADC with analog CDS block and (**b**) proposed TSSS ADC with DDA.

**Figure 7:**
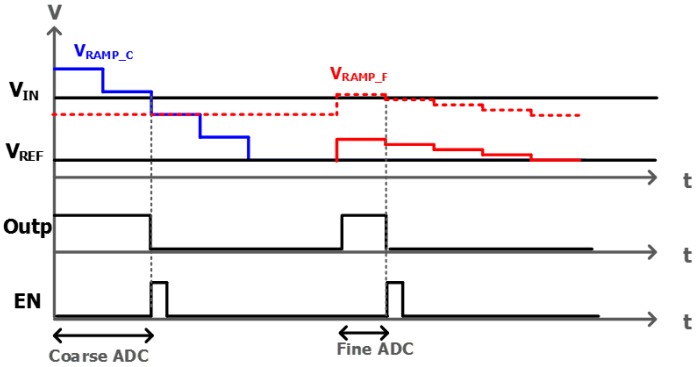
Timing diagram for proposed TSSS ADC.

Figure 6a shows a schematic of conventional TSSS ADC with an analog correlated double sampling (CDS) block. The holding capacitor (C_H_) that stores the final coarse analog voltage (V_REF_) is connected to the external ramp signals (V_RAMP_) in a series. When the next fine ramp signal drives the comparator through C_H_, the fine ramp slope would be distorted by parasitic capacitances that come from device mismatch, different metal routings, and switching noises, such as clock feed-through and charge injection. The different parasitic capacitances of each column ADC cause the different gain and linearity, finally resulting in column fixed-pattern noise (CFPN). In order to overcome such limitation in conventional TSSS ADC, we propose an alternative TSSS ADC structure as shown in Figure 6b. Figure 7 shows the timing diagram of proposed TSSS ADC with 4-input comparator that uses differential difference amplifier (DDA) for analog CDS operation. The differences of the proposed TSSS ADC from conventional one are as following. First, SADC1 and SADC2 are eliminated, leading to low offset voltage of comparator and reduced mismatch. Second, C_H_ is connected to V_RAMP_C_ which means that the fine ramp slope is not affected by the parasitic capacitances, leading to small variation of the slope of V_RAMP_F_. 

## 4. Experimental Results

### 4.1. Simulation Results and Chip Photograph

**Figure 8:**
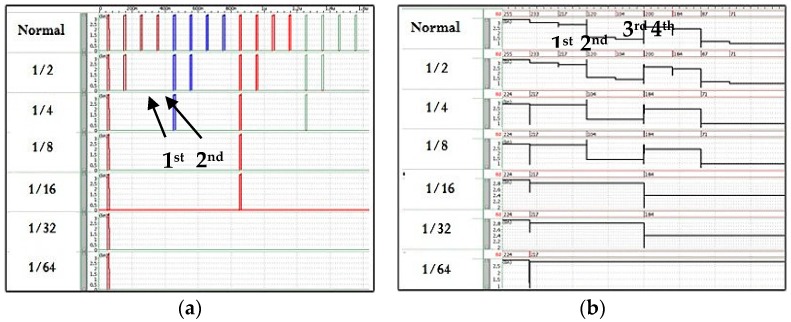
Simulation results showing (**a**) row control signals and (**b**) pixel output voltage (V_IN_) for the input of column ADC array with different resolutions.

Figure 8 shows simulation results of configurable selection of rows and the input of ADC column array with different resolutions. As shown in Figure 8a, every row is selected at normal mode (at the top waveform of Figure 8a). For the 1/2 mode, the 1st and 2nd row are selected while the 3rd and 4th row are not. With this row control, finally pixel resolution can be configurable from full resolution down to the 1/64 resolution mode depending on the situations of ISS. Like row control signals, the input of column ADC array can be configurable as shown in Figure 8b. While V_IN_ of every column is transferred from pixel array to each column ADC array, only selected column is operated with scaled pixel resolutions.

### 4.2. Measurement Results

**Figure 9:**
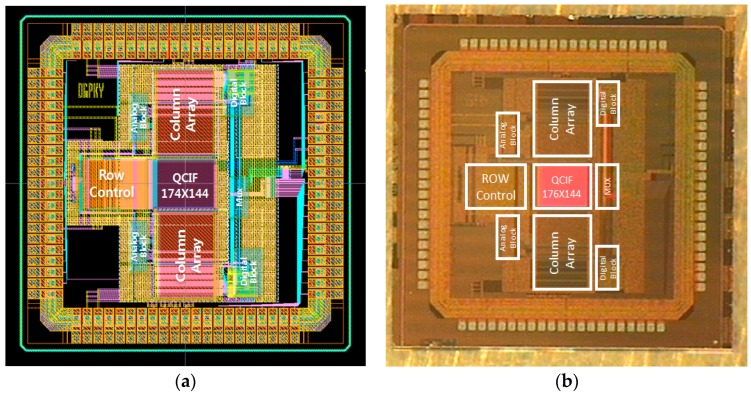
(**a**) The chip layout of the proposed CIS and (**b**) microphotograph of the fabricated CIS.

Figure 9 shows the entire layout and microphotograph of the CIS fabricated with a 0.18 μm 1-poly 4-metal CMOS process. The area of sensor core is 2.89 mm^2^ (1.7 mm × 1.7 mm) and the chip area is 5.52 mm^2^ (2.35 mm × 2.35 mm). The area of 4-shared 4T-APS in the proposed CIS is 4 μm × 4 μm. In order to minimize CFPN, all columns are arranged to have identical repetition pattern. Dummy pixels are added to each edge of pixel array to more even out the performance of effective pixels. The number of pixels is 176 × 144 (QCIF resolution) for full resolution (=the highest resolution).

The chip is attached to a PCB via a chip-on-board (COB) process. For measurement, signals for control operations are generated with a field programmable gate array (FPGA). The measurement environment is set up so that generated signals activate the CIS whose output is then stored in a register inside the FPGA. Its value is eventually sent to a computer which renders an image on its computer monitor. We observe that power consumption is drastically reduced with the decrease of resolution. Power consumption for normal, 1/2, 1/4, 1/8, 1/16, 1/32, and 1/64 modes is about 10, 5.2, 2.7, 1.6, 0.9, 0.5, and 0.3 mW, respectively.

**Figure 10:**
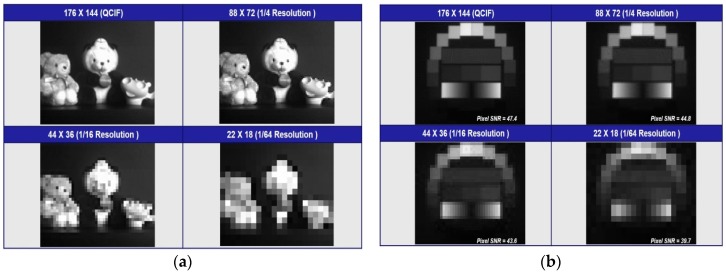
(**a**) Measured images for multi-mode pixel and (**b**) measured images for standard chart to obtain SNR.

Figure 10a shows images with different resolution modes. The one with highest resolution is shown, followed by ones with 1/4, 1/16, and 1/64 resolution respectively. Figure 10b shows the results from the analysis of testing charts as a way to analyze sample photographs for signal to noise (SNR) measurement. With the decrease of resolution from full resolution mode to 1/64 mode, SNR is reduced from 47.4 to 39.7 dB along with the decrease of power consumption from 10 to 0.3 mW. Table 1 summarizes the detailed specifications of the proposed CIS. And Table 2 summarizes the performance comparison with other works that have comparable image resolutions (such as CIF to QVGA) and technology nodes to this work. The power figure of merit (FOM) [12] is given by:

$$\mathrm{Power}\;\mathrm{FOM}=\frac{Total\;power\;consumption}{\#\;of\;pixels\;\times Frame\;rate}\left[\frac{W}{pixels\cdot fps}\right].$$

As shown in Table 2, the power FOM is relatively small and similar as that of [13,14]. However, unlike [13,14], the power consumption of CIS in our work can be reduced further with 1/64 mode, while the CIS in [13,14] maintains the constant power consumption regardless of modes (peace mode and emergency mode in ISS).

## 5. Conclusions

A CIS (with QCIF resolution) with an improved TSSS ADC that supports configurable resolutions (1, 1/2, 1/4, 1/8, 1/16, 1/32, and 1/64 resolution mode) is proposed for intelligent surveillance system applications. With the direct connection of fine ramp signals to the comparators, parasitic capacitances causing mismatch and CFPN are reduced. In addition, the measurement results show that figure of merit (FOM) of QCIF CIS is 5.524 μW·fps, which represents a lowest power consumption than ever described in the previous research. The power consumption can further be reduced from 10 to 0.3 mW by lowering the resolution mode from full resolution mode to 1/6 resolution mode. Therefore, the proposed CIS can be used for ultra-low power image sensors in consumer electronics that require always-on sensing features. 

## Figures and Tables

**Table 1:** Performance summary of the proposed CIS.

**Array Format**	QCIF (176 × 144)
**Pixel Size**	4.4 μm × 4.4 μm
**Fill Factor**	9%
**Dynamic Range**	61.8 dB
**ADC Resolution**	8-bit
**Frame Rate**	14 frame/s
**Power Supply**	3.3 V (analog)/1.8 V (digital)
**Power Consumption**	10 mW (High-resolution mode)
	0.3 mW (1/64 resolution mode)
	11.3 μW(per column)
	0.4 μW (per column @power shut off)
**SNR**	47 dB (High-resolution mode)
	39.7 dB (1/64 resolution mode)
**Area**	5.52 mm^2^ (2.35 mm× 2.35 mm)
**Process**	Towerjazz 0.18 μm CIS

**Table 2:** Performance comparison of the proposed CIS (figure of merit: FOM).

Ref.	Pixels	Technology	Frame Rate	Power	Power FOM	Read-Out
		(μm)	(fps)	(mW)	(nW/pixels∙fps)	Method
[9]	128 × 128	0.35	30	30	61.04	Single ADC (SAR ^1^)
[12]	256 × 256	0.35	30	75	38.01	Column ADC (SS ^2^)
[13]	128 × 128	0.13	9	16	108.51	In-pixel ADC
[14]	176 × 144	0.25	30	20	26.31	External ADC
[15]	320 × 240	0.35	15	30	26.04	Column ADC (SS)
This work	176 × 144	0.18	14	10	28.18	Column ADC (TSSS ^3^)
	1/64 mode		896	0.3	0.85	

^1^ Successive approximation ADC, ^2^ Single-Slope ADC, and ^3^ Two-step Single-Slope ADC.
